# Health Improvement and Educational Attainment in Secondary Schools: Complementary or Competing Priorities? Exploratory Analyses From the School Health Research Network in Wales

**DOI:** 10.1177/1090198117747659

**Published:** 2018-01-06

**Authors:** Hannah J. Littlecott, Sara Long, Jemma Hawkins, Simon Murphy, Gillian Hewitt, Gemma Eccles, Adam Fletcher, Graham F. Moore

**Affiliations:** 1 Cardiff University, Cardiff, UK; 2 Office for National Statistics, Newport, UK

**Keywords:** attendance, educational outcomes, linear regression, school environment, school health

## Abstract

*Background*. Implementing health improvement is often perceived as diverting resource away from schools’ core business, reflecting an assumption of a “zero-sum game” between health and education. There is some evidence that health behaviors may affect young people’s educational outcomes. However, associations between implementation of school health improvement and educational outcomes remains underinvestigated. *Methods*. The study linked school-level data on free school meal (FSM) entitlement, educational outcomes, and school attendance, obtained from government websites, with data from the School Environment Questionnaire (SEQ) on health improvement activity collected in Wales (2015/2016). Spearman’s rank correlation coefficients and linear regression models tested the extent of association between health improvement activity and attendance and educational outcomes. *Results*. SEQ data were provided by 100/115 network schools (87%), of whom data on educational performance were obtained from 97. The percentage of pupils entitled to FSM predicted most of the between-school variance in achievement and attendance. Linear regression models demonstrated significant positive associations of all measures of health improvement activity with attainment at Key Stage (KS) 3, apart from mental health education in the curriculum and organizational commitment to health. Student and parent involvement in planning health activities were associated with improved school attendance. There were no significant associations between health improvement and KS4 attainment. *Conclusion*. Implementing health improvement activity does not have a detrimental effect on schools’ educational performance. There is tentative evidence of the reverse, with better educational outcomes in schools with more extensive health improvement policies and practices. Further research should investigate processes by which this occurs and variations by socioeconomic status.

Schools provide an important setting for universal intervention to improve pupil health (Bonell et al., 2014; Langford et al., 2014; Moore, Littlecott, Turley, Waters, & Murphy, 2015). Health behaviors such as physical inactivity and substance use, as well as emotional well-being, often worsen during adolescence, making this a critical life-course period for intervention to improve health (Elgar et al., 2015; Hanson & Chen, 2007; Viner et al., 2012). However, due to pressures on schools to attain high levels of academic achievement, “core business” is often defined narrowly in terms of performance in core subjects such as Mathematics, Science, and English. Health issues such as curriculum time spent in Physical Education are not currently monitored by the schools’ inspectorate in England (Weiler, Allardyce, Whyte, & Stamatakis, 2013) and only make up a small part of assessments by the Welsh schools’ inspectorate. Recent evidence shows that when schools are under pressure, Health Education may be one of the first things to be discarded from the curriculum (Formby & Wolstenholme, 2012).

In this article, health improvement policies and practices are defined as actions undertaken by a school which aim to improve students’ health and well-being. These include the creation of policies, delivery of program and services, and provision of specialist staff. Bonell et al. (2014) argue that resistance from policymakers and school stakeholders to implementing health improvement policies and practices is driven by perceptions of a “zero-sum game,” whereby health improvement and educational attainment are framed as competing rather than synergistic goals. Implementation of health improvement policies and practices are often perceived as diverting resource from schools’ core business (Bonell et al., 2014; Walton, Signal, & Thomson, 2012), potentially compromising the likelihood of meeting educational performance targets. The increasingly narrow focus on academic attainment by the 2010-2015 coalition government, who removed references to well-being from the English inspection framework (Gove, 2012), was conceived as a means of avoiding distracting schools from their core mission. In Wales, current moves toward greater integration of health and well-being into the new school curriculum (Donaldson, 2015) may be seen by some as a threat to core business.

To date, a number of studies have investigated links between pupil’s health and health behaviors and their educational attainment. One recent study showed significant associations between young people’s breakfast consumption and subsequent educational attainment (Littlecott, Moore, Moore, Lyons, & Murphy, 2015). Reviews and evidence syntheses have, to date, found equivocal support regarding behaviors such as physical activity and diet and educational outcomes/attainment (Adolphus, Lawton, & Dye, 2013; Public Health England, 2013, 2014). However, while testing the “zero-sum game” hypothesis requires direct evaluations of how increasing the level of health improvement policies and practices within schools may affect, positively or negatively, educational attainment, this rarely occurs. One review of multicomponent school health improvement programs found a mixture of positive effects on, and no impairment of, educational outcomes (Murray, Low, Hollis, Cross, & Davis, 2007). However, a systematic review of Health Promoting Schools (HPS) framework interventions (Langford, Bonell, Jones, & Campbell, 2015; Langford et al., 2014) found that data on attendance or academic performance were rarely collected by public health evaluators (Langford et al., 2015; Langford et al., 2016).

The HPS approach advocates whole system change, including integration of health education into the curriculum, creation of healthy school environments, and engagement with parents and communities. Educational attainment has many other potential confounding influences, such as parenting styles (Spera, 2005) and neighborhood effects (Ainsworth, 2002). The attainment level of a school is typically explained to a large extent by the composition of its intake and not purely by what the school does (Leckie & Goldstein, 2017). However, there is clear evidence that schools have the potential to positively influence both health (Bonell et al., 2013) and attainment outcomes (Leckie & Goldstein, 2017), and understanding how these goals compete with, or complement one another, remains vital. A key tenet of settings approaches that underpin the HPS framework is the need for alignment between public health and “core business” agendas (Dooris, 2006). It therefore emphasizes the need for synergistic approaches to health and education and for holistic approaches that influence multiple health outcomes simultaneously, and work with systems beyond the school gates to support pupil well-being (World Health Organization, 1998). School-based interventions may enhance health and education via mechanisms such as cognition and sensory perceptions or through increasing school connectedness and reducing absenteeism (Basch, 2011), while one review found that schools adhering to principles consistent with the HPS framework did better in terms of health and education (Michael, Merlo, Basch, Wentzel, & Wechsler, 2015). Safe, positive school environments with high levels of engagement with families and community members in schools also had positive outcomes across health and educational domains (Michael et al., 2015). Hence, rather than detracting from attainment outcomes, a focus on health and well-being may plausibly improve attainment outcomes.

This article will explore how existing variance in the embeddedness of health improvement policies and practices in Welsh secondary school systems, in line with characteristics outlined by the HPS framework, correlates with standardized markers of educational attainment. This offers an opportunity to test the hypothesis driving resistance to the implementation of school health improvement policies and practices; that attainment will be lower in schools which dedicate greater resource to health improvement. We do not in this exploratory analysis attempt to develop detailed typologies of health improvement policies and practices that are, or are not, associated with educational outcomes, but focus on the association between the embeddedness of health improvement policies and practices and attainment outcomes.

## Method

### Sampling

This study uses data collected from the School Health Research Network (SHRN) School Environment Questionnaire (SEQ) in Wales in 2016. The SHRN is an infrastructure for school-based health improvement research in Wales. In 2016, network schools represented just over half (*N* = 115; 54.3%) of all secondary schools in Wales (*N* = 212), with representation from all 22 local authority areas. Schools were recruited to the network through three mechanisms. First, those participating in the 2013/2014 Welsh Health Behavior in School-aged Children (HBSC) Study were invited to join (60 of 82 did so). Second, nine schools in South Wales that participated in a HBSC substudy in 2013 joined the network. Finally, 44 schools joined in 2015 during a period of open recruitment. Each member school had a designated staff member who was briefed about the SEQ via emails, newsletters, and at an event for schools in June 2015. Network schools were invited to participate in the cross-sectional SEQ from January to March 2016. In line with the HPS framework (Tang et al., 2008), sections of the SEQ related to (a) health and well-being education in the curriculum (i.e., the presence of various health topics throughout the wider curriculum), (b) the school social environment in terms of policies for health and student involvement, and (c) partnerships with schools and wider communities relating to health. Within these sections, questions focused on the following health and well-being issues: physical activity, healthy eating, tobacco, drugs and alcohol, mental health and well-being, sex and relationships, health service providers, behavior and discipline, and self-harm prevention. The SEQ was mailed to each designated staff member with a request to nominate a senior management team member to complete it in paper format. Information regarding the purpose of the SEQ was provided in its introduction and completion was taken as consent. Ethical approval was obtained from the Cardiff University School of Social Sciences Research Ethics Committee.

### Measures

#### Socioeconomic Status, Educational Attainment, and Attendance

The government website mylocalschool.wales.gov.uk provides official data for each school in Wales, including data on attendance, attainment, and free school meal (FSM) entitlement. Data on the 3-year rolling average percentage of pupils entitled to FSM from the 2012/2013 to 2015/2016 school years within each school, school-level data on attendance rates, and the percentage of pupils within each school who reached the expected level in core subjects (English or Welsh, Mathematics, and Science) by the end of Key Stage 3 (KS3; 11-14 years) and Key Stage 4 (KS4; 14-16 years) were obtained. Educational outcome variables are based on nationally set thresholds. At the end of KS3, those who have reached Level 5, out of a possible range of Levels 3 to 7, in core subjects (English/Welsh, Mathematics, and Science) are deemed to have met the expected standard. At KS4, pupils are deemed to have met the expected standard if they obtain 5 or more GCSEs at Grade A* to C, including in English/Welsh and Mathematics. Attendance and attainment data were obtained for the 2012/2013 school year and the 2015/2016 school year.

#### Physical and Mental Health Education in School Curriculum

Schools were presented with a grid for each of a range of topic areas (physical activity, diet, drugs, tobacco, alcohol, sex education, and mental health) and asked to indicate which year groups received health education in that topic, and in which subject areas (Personal and Social Education or Welsh Baccalaureate, Science, Vocational courses, Other, not taught to this year group). For each item, a sumscore was generated to represent a combination of the number of subject areas, alongside the number of year groups delivered to. Sumscores were then subjected to factor analysis. Items relating to physical health (physical activity, diet, drugs, tobacco, alcohol, and sex education) demonstrated loadings greater than .4 on the first factor and formed a scale with a Cronbach’s alpha coefficient of .83, indicating good internal consistency. The single item on mental health education within the curriculum was the only item not to load onto this factor. Hence, two variables were constructed: (a) physical health education in the curriculum and (b) mental health education in the curriculum.

#### School Health Policies

Schools were asked to indicate which of a list of health and well-being areas were covered by a written policy within their school. These were food and fitness, smoking, drugs, alcohol, mental health, suicide prevention, and violence against women and girls. A score was constructed to indicate the number of areas covered by schools’ written policies.

#### Involvement of Students in Developing Health Improvement Policies

Schools were asked about student involvement in developing policies on health and well-being including smoking and tobacco use, drugs, healthy eating or food and fitness, mental health and well-being, behavior and discipline, bullying, suicide prevention and/or post suicide care, sex and relationships, and violence against women and girls. Responses options included consultation with school council, consultation with other student voice groups, wider consultation with students, suggestion boxes, and other. A variable was created by summing the number of policy areas in which schools reportedly involved students (i.e., any versus no student involvement in each policy area).

#### Involvement of Parents in Health Improvement Policies and Practices

Schools were asked to estimate the proportion of parents involved in health improvement activities, with four options ranging from none to all. Schools who stated that at least some parents were involved in health improvement policies and practices were also asked in what areas parents were involved (deciding on health priority areas, delivery of health education, development of school health policies, and other), and what mechanisms were used to involve parents (PTA meetings, parent information evenings, parental questionnaires, involvement initiated by parents, through parent governors, and in one-to-one meetings). Three items were derived and subjected to factor analysis, including the proportion of parents involved in health improvement policies and practices, the number of areas in which parents were involved, and the number of mechanisms for involving parents. All items loaded onto a single factor (>.6), and formed a scale with an alpha coefficient of .66, indicating acceptable internal consistency.

#### Partnerships

Schools were asked to indicate any formal or informal partnerships that went beyond statutory requirements to help students remain or become physically active (with families, other schools, local community groups, professional sports clubs, national sport bodies, private sector businesses or organizations, local authority sports development officers, the local health board, or other). The presence of a partnership indicated for each of these nine options was given a score of one. Partnerships were used as a proxy for how well networked schools were.

#### Organizational Commitment to Student Health

Schools were asked to select up to four areas that had been prioritized by the senior management team in the past 2 academic years from a list of 10 areas to represent potential health- and non–health-related priority areas for schools. These included student emotional and mental health, student physical health, and staff health as well as items on educational performance and school environment. Scoring related to whether schools had indicated that neither, either, or both items relating to (a) student emotional/mental health and (b) student physical health were among the top four priority areas for their school from a list of 10 options (i.e., that either 0, 1, or 2 out of the four priority areas selected related to health and well-being). For each school, a score of 0 was assigned if neither student health item was selected within the four areas, “1” if one was, and “2” if both were. Schools were also asked if they had an overall written action plan for student health, and how often this was reviewed. A score of 0 was assigned if there was no action plan, 1 for action plans that were reviewed less than once a year, and 2 if there was a written action plan that was reviewed annually. These items were summed to form an ordinal scale scored from 0 (lowest level of organizational commitment to health) to 4 (highest level of organizational commitment to health). Further details of the items included within this measure are available in a previous open access article (Moore, Littlecott, Fletcher, Hewitt, & Murphy, 2016).

#### Overall Embeddedness of Health Improvement Policies and Practices

A composite measure of the embeddedness of health improvement policies and practices, in line with the Health Promoting Schools Framework, was derived through summing items for health education (physical and mental health in the school curriculum), school ethos (written policies and student involvement), and engaging family and community (parental involvement and partnerships) after scaling each from 0 to 1 by dividing by the maximum score (thus, giving equal weight to all three dimensions), such that a score of 0 indicated lowest possible embeddedness of health improvement policies and practices and 3 the highest possible embeddedness. As a sensitivity analysis, we also calculated this variable using standardized scores for each of its components before summing. The final variables produced by either methods were almost perfectly correlated with one another (*r* = .95), and predicted the same level of variance in outcomes. Hence, for ease of reading, we present only the first version of the variable.

### Statistical Analysis

First, Spearman’s rank correlation coefficients were used to examine unadjusted associations between all variables. Subsequently, linear regression analyses tested the association of health improvement variables with educational performance outcomes after adjustment for FSM entitlement. Regression models are presented without adjustment for 2012/2013 educational performance data, and with adjustment. Adjusted models are used to attempt to account for reverse causality (i.e., that schools with higher levels of attainment were more likely to adopt health improvement policies and practices) by taking account of historical differences between schools in educational performance. For KS3 analysis, due to skewness in educational attainment data, arising largely from two slight outliers, analyses were run in two additional ways as sensitivity analyses: (a) excluding two outliers and (b) using a square root transformation to reduce skewness. Findings were consistent across all methods.

## Results

### Response Rate

Out of 115 member schools, a response was received from 100 (87%), representing approximately 45% of all secondary schools in Wales. Educational attainment data were obtained for 97 out of the 100 schools. Three independent schools were excluded, due to absence of standardized data on educational attainment. Participating schools were representative of all state maintained secondary schools in Wales in terms of FSM entitlement (*M* = 16.9%; *SD* = 9.2%), school size (*M* = 907.4; *SD* = 356.8), and the percentage of young people achieving the expected level at KS3 (*M* = 88.1; *SD* = 6.5).

### Bivariate Associations

Means and ranges for all variables are presented in Table 1, while unadjusted associations between all variables of interest are presented in Table 2. There were significantly fewer written policies in more deprived schools, though a nonsignificant trend toward greater embeddedness of health education into the curriculum in more deprived schools. Results also show a nonsignificant trend toward lower student involvement in more deprived schools. There was no association between FSM entitlement and the composite measure of embeddedness of health into the school system, number of partnerships, or parental involvement. A higher level of organizational commitment to health was associated with a higher number of topic areas covered by written policies, greater parental involvement in health improvement policies and practices, greater student involvement, greater partnerships, and greater overall embeddedness of health into the school system. Organizational commitment was not significantly correlated with attainment or attendance outcomes.

**Table 1.** Mean and Range for All Variables.

Variable	Mean	Range
Free school meal entitlement	16.9	3.7-49.9
Organizational commitment	1.9	0-4
Physical health education in curriculum	35.9	11-75
Mental health education in curriculum	4.8	0-10
Written policies	4.3	0-7
Parental involvement	4.3	0-12
Student involvement	4.2	0-9
Partnerships	3.2	0-8
Overall embeddedness of health	1.4	0.31-2.43
Key Stage 3 2013	77.8	27.3-92.9
Key Stage 4 2013	54.4	25.4-77.3
Attendance 2013	92.8	87.6-95.6
Key Stage 3 2016	88.1	59.8-97.5
Key Stage 4 2016	59.0	25.2-86.8
Attendance 2016	94.3	89.8-97.3

**Table 2.** Unadjusted Associations Between Variables.^a^

Variable	Free School meal entitlement	Organizational commitment	Physical health education in curriculum	Mental health education in curriculum	Written policies	Parental involvement	Student involvement	Partnerships	Overall embeddedness of health	Key Stage 3 2013	Key Stage 4 2013	Attendance 2013	Key Stage 3 2016	Key Stage 4 2016	Attendance 2016
Free school meal entitlement	—														
Organizational commitment	−0.18	—													
Physical health education in curriculum	0.15	0.08	—												
Mental health education in curriculum	0.17	0.13	**0.28**	—											
Written policies	−**0.25**	**0.30**	**0.21**	0.12	—										
Parental involvement	0.01	**0.38**	**0.22**	**0.22**	0.10	—									
Student involvement	−0.19	**0.35**	0.15	**0.21**	0.05	**0.41**	—								
Partnerships	−0.05	**0.34**	**0.20**	**0.23**	0.16	**0.37**	**0.27**	—							
Overall embeddedness of health	−0.09	**0.49**	**0.49**	**0.59**	**0.43**	**0.67**	**0.61**	**0.60**	—						
Key Stage 3 2013	−**0.75**	0.15	−0.13	−**0.24**	0.14	−0.08	0.16	−0.10	−0.04	—					
Key Stage 4 2013	−**0.84**	0.19	−0.14	−0.18	0.15	−0.05	**0.25**	−0.04	0.05	**0.70**	—				
Attendance 2013	−**0.66**	0.09	−0.14	−0.02	**0.21**	−0.01	0.04	−0.16	0.01	**0.61**	**0.59**	—			
Key Stage 3 2016	−**0.76**	0.17	0.01	−0.04	**0.32**	**0.22**	**0.28**	0.14	**0.27**	**0.59**	**0.59**	**0.60**	—		
Key Stage 4 2016	−**0.76**	0.14	0.02	−0.06	0.16	0.10	**0.25**	−0.02	0.17	**0.63**	**0.71**	**0.56**	**0.71**	—	
Attendance 2016	−**0.72**	0.13	−0.15	−0.01	**0.21**	0.09	0.17	−0.09	0.09	**0.60**	**0.61**	**0.71**	**0.76**	**0.74**	—

a Boldface type indicates p < .05

Strong correlations of FSM with achievement and attendance indicated that the majority of variance in attainment between schools was associated with schools’ socioeconomic intake. There were significant positive associations of written health policies with KS3 educational performance in 2015/2016 and school attendance. Parental involvement and overall embeddedness of health improvement policies and practices were also significantly positively associated with KS3 educational performance in 2015/2016. Student involvement was significantly positively associated with KS3 and KS4 educational performance in 2015/2016 and KS4 educational performance in 2012/2013. These figures are consistent with a hypothesis that health improvement policies and practices are associated with better educational attainment, particularly for younger students. Notably, there was no evidence that schools with higher levels of health improvement policies and practices performed better educationally in 2012/2013, apart from KS4 student involvement. Hence, data are not consistent with a hypothesis of reverse causality (that schools who are performing better educationally adopt more health improvement policies and practices).

### Multivariate Analyses

#### Key Stage 3 (11-14 Years)

Regression analyses presented in Table 3 indicate that, after adjustment for FSM entitlement, KS3 educational attainment was significantly and positively associated with overall embeddedness of health into the school system, embeddedness of physical health education in the curriculum, coverage of health and well-being within written policies, parental involvement in health improvement policies and practices, and student involvement in developing health policies and partnerships. Mental health education in the curriculum and organizational commitment to health were not associated with educational attainment. Most of the variance in educational attainment was associated with FSM entitlement (*R*^2^ = 0.60), with no additional variance explained by 2013 attainment. Once the composite measure of embeddedness of health was included within the model, the proportion of variance explained increased to approximately two thirds (*R*^2^ = 0.67).

**Table 3.** B-Coefficients and 95% Confidence Intervals From Linear Regression Analyses Examining Associations With Educational Attainment and Attendance With and Without Adjustment for 2013 Attainment and Attendance Data.^a^

Variables	Coefficient (95% CIs)		
	Attainment Key Stage 3 (KS3)	Attainment Key Stage 4 (KS4)	Attendance
Physical Health in Curriculum (*N* = 97)			
Unadjusted			
FSM 2016	**−.57 (−.66, –.48)**	**−1.01 (−1.19, −.84)**	**−.09 (−.11, −.07)**
Physical health education in curriculum	**.08 (.01, .14)**	**.13 (.00, .25)**	**−**.00 (**−**.01, .01)
Adjusted			
KS3 2013/KS4 2013/Attendance 2013	.02 (**−**.07, .12)	**.35 (.12, .57)**	**.39 (.25, .53)**
FSM 2016	**−.55 (–.66, –.44)**	**−.63 (−.93, −.33)**	**−.05 (−.07, −.03)**
Physical health education in curriculum	**.08 (.01, .14)**	.11 (**−**.01, .23)	**−**.00 (**−**.01, .01)
Mental Health in Curriculum (*N* = 97)			
Unadjusted			
FSM 2016	**−.56 (–.66, −.47)**	**−1.00 (−1.18, −.82)**	**−.09 (−.11, −.07)**
Mental health education in curriculum	.25 (**−**.08, .59)	.26 (**−**.40, .92)	.05 (**−**.01, .11)
Adjusted			
KS3 2013/KS4 2013/Attendance 2013	.05 (**−**.05, .14)	**.37 (.14, .60)**	**.38 (.24, .52)**
FSM 2016	**−.53 (−.65, −.42)**	**−.59 (−.89, −.29)**	**−.05 (−.07, −.03)**
Mental health education in curriculum	.28 (**−**.06, .63)	.29 (**−**.34, .91)	.03 (**−**.03, .08)
Written Policies (*N* = 97)			
Unadjusted			
FSM 2016	**−.53 (−.63, –.44)**	**−.98 (−1.16, −.80)**	**−.09 (−.11, −.07)**
Written policies	**.60 (.07, 1.13)**	.29 (**−**.76, 1.34)	.03 (**−**.07, .13)
Adjusted			
KS3 2013/KS4 2013/Attendance 2013	.03 (**−**.07, .13)	**.37 (.14, .60)**	**.39 (.25, .53)**
FSM 2016	**−.51 (−.63, −.40)**	**−.57 (−.88, −.27)**	**−.05 (−.07, −.03)**
Written policies	**.60 (.07, 1.13)**	.27 (**−**.73, 1.27)	.01 (**−**.08, .10)
Parental Involvement (*N* = 97)			
Unadjusted			
FSM 2016	**−.56 (–.64, –.47)**	**−.99 (–1.17, –.81)**	**−.09 (−.11, −.07)**
Parental involvement	**.50 (.24, .76)**	.39 (**−**.15, .93)	.05 (**−**.00, .10)
Adjusted			
KS3 2013/KS4 2013/Attendance 2013	.04 (**−**.05, .13)	**.38 (.15, .60)**	**.39 (.26, .53)**
FSM 2016	**−.53 (−.64, −.43)**	**−.58 (−.88, −.28)**	**−.05 (−.07, −.03)**
Parental involvement	**.50 (.24, .77)**	.43 (**−**.09, .94)	**.05 (.01, .10)**
Student Involvement (*N* = 97)			
Unadjusted			
FSM 2016	**−.53 (−.62, −.44)**	**−.95 (−1.13, −.77)**	**−.09 (−.10, −.07)**
Student involvement	**.49 (.16, .81)**	.60 (**−**.05, 1.25)	.05 (**−**.02, .11)
Adjusted			
KS3 2013/KS4 2013/Attendance 2013	.03 (**−**.07, .12)	**.34 (.11, .57)**	**.40 (.27, .54)**
FSM 2016	**−.51 (−.62, −.40)**	**−.59 (−.90, −.29)**	**−.04 (−.06, −.02)**
Student involvement	**.49 (.16, .81)**	.40 (**−**.24, 1.04)	**.06 (.01, .12)**
Partnerships (*N* = 97)			
Unadjusted			
FSM 2016	**−.56 (−.65, −.47)**	**−.98 (−1.16, −.80)**	**−.09 (−.11, −.07)**
Partnerships	.57 (.07, 1.06)	**−**.12 (**−**1.11, .87)	**−**.04 (**−**.13, .06)
Adjusted			
KS3 2013/KS4 2013/Attendance 2013	.05 (**−**.04, .15)	**.37 (.14, .60)**	**.39 (.25, .53)**
FSM 2016	**−.53 (−.64, −.42)**	**−.58 (−.89, −.28)**	**−.05 (−.07, −.03)**
Partnerships	**.62 (.11, 1.12)**	**−**.04 (**−**.99, .91)	.00 (**−**.08, .09)
Overall Embeddedness (*N* = 97)			
Unadjusted			
FSM 2016	**−.55 (−.64, −.47)**	**−.99 (−1.16, −.81)**	**−.09 (−.11, −.07)**
Overall embeddedness	**4.17 (2.35, 5.98)**	3.56 (–.28, 7.41)	.27 (–.11, .65)
Adjusted			
KS3 2013/KS4 2013/Attendance 2013	.05 (**−**.04, .14)	**.36 (.13, .58)**	**.39 (.25, .53)**
FSM 2016	**−.52 (−.62, −.42)**	**−.60 (−.90, −.30)**	**−.05 (−.07, −.03)**
Overall embeddedness	**4.27 (2.44, 6.09)**	3.18 (**−**.50, 6.86)	.28 (**−**.05, .61)
Organizational Commitment (*N* = 97)			
Unadjusted			
FSM 2016	**−.55 (−.64, −.45)**	**−.98 (−1.16, −.80)**	**−.09 (−.11, −.07)**
Organizational commitment	.39 (**−**.29, 1.06)	.30 (**−**1.02, 1.63)	.06 (**−**.07, .19)
Adjusted			
KS3 2013/KS4 2013/Attendance 2013	.03 (**−**.07, .12)	**.37 (.14, .60)**	**.39 (.25, .52)**
FSM 2016	**−.53 (−.64, −.42)**	**−.58 (−.88, −.28)**	**−.05 (−.06, −.02)**
Organizational commitment	.38 (**−**.30, 1.06)	**−**.07 (**−**1.35, 1.22)	.05 (**−**.06, .17)

Note. FSM = free school meal.

a Boldface type indicates *p* < .05.

#### Key Stage 4 (14-16 Years)

Regression analyses presented in Table 3 indicate that, after adjustment for FSM entitlement, KS4 educational attainment was not significantly associated with any health improvement policies and practices. Prior to this adjustment, a significant association was observed with physical health education in the curriculum. Variance in educational attainment was associated with both FSM entitlement and 2013 KS4 educational attainment. Except for partnerships and organizational commitment, which had marginal negative coefficients, all coefficients for associations between health improvement policies and practices and KS4 performance were in a positive direction, and hence are inconsistent with a hypothesis of negative impact of health improvement policies and practices on attendance. Most of variance in educational attainment was associated with FSM entitlement (*R*^2^ = 0.61), with a further 7% of explained by 2012/2013 attainment (*r* = .68). Addition of the HPS variable did not lead to any further increase in the *R*^2^ squared value.

#### Attendance (Whole School)

Parental and student involvement were significantly associated with attendance after adjustment for 2012/2013 attendance data, though there were no other significant predictors of attendance. All coefficients for associations between health improvement policies and practices and attendance were in a positive direction, and hence are inconsistent with a hypothesis of negative impact of health improvement policies and practices on attendance. Approximately half of the variance in attendance was associated with FSM entitlement (*R*^2^ = 0.53), increasing only slightly after adjustment for 2013 attendance rates (*r* = .57). There was no further increase after addition of the composite measure of embeddedness of health.

## Discussion

Overall, this article found no support for the hypothesis that increased health improvement policies and practices within schools compromises educational performance. Hence, concerns that have driven an increasingly narrow focus on educational metrics in some jurisdictions such as England (Bonell et al., 2014), and which to date have been largely untested by public health researchers (Langford et al., 2015), appear to be unfounded. Indeed, there was some evidence of the opposite within the younger age groups; schools with a higher emphasis on pupils’ health and well-being tended to do better educationally, after adjustment for socioeconomic differences and historical attainment differences between schools. Results demonstrated a significant association between KS3 educational attainment and embeddedness of health into the school system. No evidence of a link between health improvement policies and practices and attendance or KS4 educational attainment was observed, apart from small significant associations between student and parent involvement and attendance. However, all coefficients, while nonsignificant, were in a positive direction, thus providing no suggestion of any detrimental effect of health improvement policies and practices on KS4 attainment or attendance.

The lack of association observed between educational outcomes at KS4 (age 14-16 years) and health improvement policies and practices may be due to a decrease in the influence of the school on students’ lives as they get older, in line with the established decrease in parental influence (Aveyard et al., 2004). For example, Aveyard et al. (2004) found that, when analyzed by year group, value added by schools explained a higher percentage of the variance in smoking within the younger year groups. It may also however be due to differences in assessment method, with KS3 outcomes based on teacher assessment of pupil performance, as opposed to the use of qualifications at the KS4 metric. The majority of variance between schools in terms of attainment appeared to be explained by the socioeconomic composition of their intake. Pupil health outcomes are also typically patterned by both school and family-level socioeconomic status (Moore & Littlecott, 2015), while there is evidence that school health improvement policies and practices can reduce, or increase, inequalities in health depending on the nature of intervention (Moore et al., 2015). There is therefore a need for further analyses in order to understand what role the embeddedness of health into schools may play in reducing, or increasing, inequalities in educational attainment.

### Strengths and Limitations

This study uses a large, sample of secondary schools in Wales and capitalizes on routinely available data to present strong evidence of an association between educational outcomes and embeddedness of health within schools. However, while this sample is representative of Welsh schools in terms of measured variables, included educational attainment, attendance, school size, and free school meal entitlement, there may be unmeasured differences between those schools who are members of the School Health Research Network and those who are not in terms of their approaches to health improvement policies and practices. Data on school-improvement practices are based on self-report, while reports captured the quantity rather than quality of health improvement policies and practices. Some additional activities within parent and community involvement may not have been captured. Moreover, partnerships data were only available in relation to physical activity, which may be an imprecise proxy for a school’s connectedness to their communities more generally. Student and parental involvement in health improvement policies and practices might reflect a broader tendency toward involvement of students in decision making within the school. Hence, it may be that student and parental involvement more broadly is predictive of attainment, rather than involvement specifically in health. Furthermore, educational attainment data were available at the school-level only. While we have attempted to mitigate the potential for reverse causality by the inclusion of historical educational performance within regression models, causal inferences cannot be firmly established. Moreover, routine data did not allow for disaggregation of authorized and unauthorized absence and, in the case of KS3 attainment data, attendance, and FSM entitlement, relied on accurate record keeping by schools.

### Implications

Nevertheless, this study provides possibly the strongest evidence to date that the implementation of school health improvement policies and practices does not have a detrimental effect on students’ educational outcomes. To the contrary, there is reason to believe that greater embeddedness of health improvement policies and practices in schools may represent a means of improving educational outcomes. These findings support an emerging body of theory and evidence that can help persuade schools and educational policymakers that implementing health improvement policies and practices will not have a detrimental effect on students’ educational attainment (Bonell et al., 2014) and that a Health Promoting Schools approach should be advocated. Moves toward an increasingly narrow focus on educational metrics in some jurisdictions appear to have been misguided, while there should be few fears that movements toward increased focus on health and well-being in countries such as Wales will detract from schools’ core business. In addition, findings indicate that a focus on student and parent involvement may present a strategy for schools to increase student attendance rates, although the mechanisms by which this may result in improved educational attainment should be a focus of future research. Aspects of health improvement intervention may work synergistically with educational outcomes through impacts on cognitive functioning (Basch, 2011), an effect on school culture (Bonell et al., 2014), school connectedness (Chapman, Buckley, Sheehan, & Shochet, 2013; Jamal et al., 2013), and building positive relationships between staff and students (Moore et al., 2016). This exploratory analysis did not attempt to develop detailed typologies of health improvement policies and practices that were, or were not, associated with educational outcomes, or underpinning mechanisms. With expansion of the School Health Research Network to include most schools in Wales, our follow-up surveys will provide a larger sample of schools with whom such analyses may be conducted. Given the substantial role of socioeconomic status in determining pupil outcomes, and prior evidence of mixed effects of health improvement interventions on socioeconomic inequality in health and well-being, future analyses should focus on the role of health improvement policies and practices in reducing or exacerbating socioeconomic inequalities in educational attainment. Further analyses linking school environment data to individual-level health and well-being and attainment outcomes is important in unpacking the potential mechanisms linking health improvement policies and practices to educational attainment.
